# Supplementary material for: Illuminating the mechanism and allosteric behavior of NanoLuc luciferase
Source: Nat Commun. 2023 Nov 29;14:7864. doi: 10.1038/s41467-023-43403-y (PMC10687086; doi:10.1038/s41467-023-43403-y)
Supplement: Supplementary file 3 — Description of Additional Supplementary Files [file 41467_2023_43403_MOESM3_ESM.pdf]

Description of Additional Supplementary File

Supplementary Movie 1. The visualization of the ASMD simulation of pulling CTZ luciferin (orange sticks) inside of NanoLuc^{CTZ} (light pink cartoon; side chains as lines). The side chains of H93, Y94, Y109, F110, Y114, D139, and R162 are shown as thin pink sticks.

Supplementary Movie 2. The visualization of the ASMD simulation of pulling CTZ luciferin (orange sticks) inside the open beta-barrel form of NanoLuc (yellow cartoon; side chains as lines). The side chains of H93, Y94, Y109, F110, Y114, D139, and R162 are shown as thin pink sticks.

Supplementary Movie 3. The visualization of the ASMD simulation of pulling CTZ luciferin (orange sticks) inside the closed beta-barrel conformation of NanoLuc (cyan cartoon; side chains as lines). The side chains of H93, Y94, Y109, F110, Y114, D139, and R162 are shown as thin pink sticks.
