# Supplementary material for: Illuminating the mechanism and allosteric behavior of NanoLuc luciferase
Source: Nat Commun. 2023 Nov 29;14:7864. doi: 10.1038/s41467-023-43403-y (PMC10687086; doi:10.1038/s41467-023-43403-y)
Supplement: Supplementary file 7 — Reporting Summary [file 41467_2023_43403_MOESM7_ESM.pdf]

nature portfolio

Corresponding author(s): David Bednar, Yves L. Janin, Martin Marek

Last updated by author(s): Sep 11, 2023

Reporting Summary

Nature Portfolio wishes to improve the reproducibility of the work that we publish. This form provides structure for consistency and transparency in reporting. For further information on Nature Portfolio policies, see our Editorial Policies and the Editorial Policy Checklist.

Statistics

For all statistical analyses, confirm that the following items are present in the figure legend, table legend, main text, or Methods section.

n/a	Confirmed	
☐	☒	The exact sample size (*n*) for each experimental group/condition, given as a discrete number and unit of measurement
☐	☒	A statement on whether measurements were taken from distinct samples or whether the same sample was measured repeatedly
☒	☐	The statistical test(s) used AND whether they are one- or two-sided<br>*Only common tests should be described solely by name; describe more complex techniques in the Methods section.*
☒	☐	A description of all covariates tested
☒	☐	A description of any assumptions or corrections, such as tests of normality and adjustment for multiple comparisons
☐	☒	A full description of the statistical parameters including central tendency (e.g. means) or other basic estimates (e.g. regression coefficient) AND variation (e.g. standard deviation) or associated estimates of uncertainty (e.g. confidence intervals)
☒	☐	For null hypothesis testing, the test statistic (e.g. *F*, *t*, *r*) with confidence intervals, effect sizes, degrees of freedom and *P* value noted<br>*Give P values as exact values whenever suitable.*
☒	☐	For Bayesian analysis, information on the choice of priors and Markov chain Monte Carlo settings
☒	☐	For hierarchical and complex designs, identification of the appropriate level for tests and full reporting of outcomes
☒	☐	Estimates of effect sizes (e.g. Cohen's *d*, Pearson's *r*), indicating how they were calculated

Our web collection on statistics for biologists contains articles on many of the points above.

Software and code

Policy information about availability of computer code

Data collection	Avogadro 1.2.0, RESP ESP charge Derive (R.E.D.) Server Development 2.0, MGLTools 1.5.4, RCSB PDB, H++ web server v. 4.0, AutoDock Vina 1.1.2, HotSpot Wizard 3.1, AmberTools16, High Throughput Molecular Dynamics (HTMD) 2, PROPKA 2.0, Amber 16
Data analysis	KinTek Global Kinetic Explorer 6.3.170707, GraphPad Prism 8.4.3, XDS version January 31, 2020, CCP4 7.0.073 (including Aimless), Phenix 1.19.2-4158 (including Phaser MR, Phenix.refine, eLBOW), COOT 0.8.9.2, PyMOL 1.8.4, Excel 2209, Origin 6.1, VMD 1.9.3

For manuscripts utilizing custom algorithms or software that are central to the research but not yet described in published literature, software must be made available to editors and reviewers. We strongly encourage code deposition in a community repository (e.g. GitHub). See the Nature Portfolio guidelines for submitting code & software for further information.

Data

Policy information about availability of data

All manuscripts must include a data availability statement. This statement should provide the following information, where applicable:

- Accession codes, unique identifiers, or web links for publicly available datasets
- A description of any restrictions on data availability
- For clinical datasets or third party data, please ensure that the statement adheres to our policy

Atomic coordinates and structural factors have been saved in the Protein Data bank under PDB ID accession codes: 8AQH, 8AQI, 8AQ6 and 8BO9. The authors will

release the atomic coordinates and experimental data upon article publication. The primary data from molecular dynamics simulations are available in Zenodo repository with the identifier www.doi.org/10.5281/zenodo.8302143. Source data are provided with this paper.

Research involving human participants, their data, or biological material

Policy information about studies with human participants or human data. See also policy information about sex, gender (identity/presentation), and sexual orientation and race, ethnicity and racism.

Reporting on sex and gender	n/a
Reporting on race, ethnicity, or other socially relevant groupings	n/a
Population characteristics	n/a
Recruitment	n/a
Ethics oversight	n/a

Note that full information on the approval of the study protocol must also be provided in the manuscript.

Field-specific reporting

Please select the one below that is the best fit for your research. If you are not sure, read the appropriate sections before making your selection.

☒ Life sciences ☐ Behavioural & social sciences ☐ Ecological, evolutionary & environmental sciences

For a reference copy of the document with all sections, see nature.com/documents/nr-reporting-summary-flat.pdf

Life sciences study design

All studies must disclose on these points even when the disclosure is negative.

Sample size	The displayed mean values and their respective SDs were obtained from bootstrapping analysis of the calculation of kinetics. We used randomly selected 80 % of the data and repeated the calculation 100 times, which are the standard settings of the bootstrapping analysis.
Data exclusions	No data were exluded from the analysis.
Replication	All biochemistry experiments were done at least with three independent replicas. All attempts at replication were successful.
Randomization	he displayed mean values and their respective SDs were obtained from bootstrapping analysis of the calculation of kinetics. We used randomly selected 80 % of the data and repeated the calculation 100 times, which are the standard settings of the bootstrapping analysis.
Blinding	We used randomly selected 80 % of the data and repeated the calculation 100 times, which are the standard settings of the bootstrapping analysis.

Reporting for specific materials, systems and methods

We require information from authors about some types of materials, experimental systems and methods used in many studies. Here, indicate whether each material, system or method listed is relevant to your study. If you are not sure if a list item applies to your research, read the appropriate section before selecting a response.

Materials & experimental systems

n/a	Involved in the study	
☐	☒	Antibodies
☐	☒	Eukaryotic cell lines
☒	☐	Palaeontology and archaeology
☒	☐	Animals and other organisms
☒	☐	Clinical data
☒	☐	Dual use research of concern
☒	☐	Plants

Methods

n/a	Involved in the study	
☒	☐	ChIP-seq
☒	☐	Flow cytometry
☒	☐	MRI-based neuroimaging

Antibodies

Antibodies used	Anti-NanoLuc® Monoclonal Antibody, Manufacturer: Promega, Cat. Num. N7000, lot: 0000555316; Anti-β-ACTIN Monoclonal Antibody, Manufacturer: Cell Signaling Technology, Cat. Num.: #4970S, lot: 12; Anti-rabbit IgG, HRP-linked Antibody, Manufacturer: Cell Signaling Technology, Cat. Num.: #7074S, lot: 26; Anti-mouse IgG, HRP-linked Antibody, Manufacturer: Cell Signaling Technology, Cat. Num.: #7076P2, lot: 36
Validation	For NanoLuc antibody: Validation was done by the manufacturer using Western blot assay (NanoLuc fusion proteins expressed in HEK293 cells) and Immunofluorescence (CDK2-NanoLuc fusion protein expressed in HeLa Cells). For β-ACTIN antibody: Validation was done by the manufacturer using Western blot assay (HeLa, ACTA,whole cell lysates) and Immunohistochemical analysis of paraffin-embedded human heart. For #7074S and #7076P2 - validation was done by the manufacturer.

Eukaryotic cell lines

Policy information about cell lines and Sex and Gender in Research

Cell line source(s)	ATCC (https://www.atcc.org/)
Authentication	Cell line authentication was initially performed by ATCC. Further authentication was performed by microscopy, as all cell lines used in this study (ARPE-19, HEK293T) have quite distinct morphology. Additionally, HEK293T cell line was used only for the production of lentiviral particles.
Mycoplasma contamination	The cell lines were negatively tested for mycoplasma.
Commonly misidentified lines (See ICLAC register)	No commonly misidentified cell lines were used in this study
